# Reducing Local Scouring at Bridge Piles Using Collars and Geobags

**DOI:** 10.1155/2014/128635

**Published:** 2014-08-26

**Authors:** Shatirah Akib, Noor Liana Mamat, Hossein Basser, Afshin Jahangirzadeh

**Affiliations:** Department of Civil Engineering, Faculty of Engineering, University of Malaya, 50603 Kuala Lumpur, Malaysia

## Abstract

The present study examines the use of collars and geobags for reducing local scour around bridge piles. The efficiency of collars and geobags was studied experimentally. The data from the experiments were compared with data from earlier studies on the use of single piles with a collar and with a geobag. The results showed that using a combination of a steel collar and a geobag yields the most significant scour reduction for the front and rear piles, respectively. Moreover, the independent steel collar showed better efficiency than the independent geobag below the sediment level around the bridge piles.

## 1. Introduction

Recent scour-related bridge catastrophes throughout the world have received great attention [1, 2]. Scour is local lowering of streambed elevation that takes place around structures that are constructed in flowing water. Usually, scour may occur during floods, and it can make bridges collapse [3]. Since the 1920s, Malaysia has experienced major floods during seasonal monsoons, causing a large concentration of surface-water runoff that exceeds the capacities of most rivers. States located on the east coast of Peninsular Malaysia such as Kelantan, Terengganu, Pahang, and Johor are affected significantly by massive, seasonal floods [4, 5]. Researchers have studied the problem of local scouring extensively from different points of view and under different conditions. It is well documented that the main cause of concern regarding the stability of a bridge's foundation is the occurrence of scour around the piers [3]. Several researchers have introduced different methods of reducing scour and its effects, Posey [6], Odgaard and Wang [7], Graziano et al. [8], Chiew [9], Bertoldi and Kilgore [10], and Mccorquodale and Mccorquodale [11], Parola [12], Jones et al. [13], Melville and Hadfield [14], Sarkar and Ratha [15], Akib et al. [16], Jahangirzadeh et al. [17], and others. These countermeasures for local scour at bridge piers can be grouped into two categories, that is, armoring devices and flow altering devices. Armoring devices include cable-tied blocks, tetrapods, dolos, placed riprap rocks, flexible mattresses, grout mats and bags (which are fabricated from geotextiles and filled with grout in situ), anchors (used in conjunction with mats and cable-tied blocks), and high density particles around the piers' foundations. Flow altering devices that have been used to protect piers against local scour include sacrificial piles placed upstream of the pier, Iowa vanes, and flow deflectors such collars and slots [18]. This study addresses the effectiveness of collars and geobags around bridge's piers.

The primary objective of this study was to determine the scouring action on a model of a bridge's pile using the countermeasures of steel, aluminum, and a Perspex collar, and a geobag filled with crushed concrete and palm shells. A second objective was to investigate the effect of time and both single and combined countermeasures on the development of scouring. The scour reduction efficiency of collars was established in earlier studies by Chabert and Engeldinger [19], Tanaka and Yano [20], Neill et al. [21], Ettema [22], Kumar et al. [23], Zarrati et al. [24], Jahangirzade et al. [25], and Jahangirzadeh et al. [26]. The scour reduction efficiency of geobags was investigated by Korkut et al. [27] and Akib et al. [28]. Collars also have been used in combination with other methods [29–33].

Despite the efforts of previous researchers, no studies have been conducted on the combination of a geobag and a collar to control scour around bridge piles. In the present study, we considered the effects of collars made of different materials (i.e., steel, aluminum, and Perspex) and a geobag filled with crushed concrete containing oil palm shells around bridge piles with clear-water conditions. The results of this study can be used by researchers and engineers as the basis for designing and performing future research projects in this area.

## 2. Mechanism of Scouring and the Effects of Collars and Geobags

The flow pattern and mechanisms of scouring around a bridge pile are very complex and have been reported by various investigators [19, 34–36]. Local scour around a solid pile results from the downflow of water at the upstream face of the pile and at the horseshoe vortex (HSV) at the base of the pile. Separation of the flow at the sides of the pile also creates so-called “wake vortices” which are unstable and shed alternatively from each side of the pile. They act as little tornadoes lifting the sediment from the bed and forming a scour hole downstream of the pile.

In order to protect bridge piers against scouring, different methods and countermeasures have been used by researchers. The proposed methods can be grouped broadly under two distinct categories, that is, armoring and flow altering countermeasures [37].

A collar is a type of flow altering countermeasures which controls scouring around piers by diverting the downflow of water. A collar at any level above the river bed divides the flow into two regions above and below the collar. For the region above the collar, the countermeasure acts as an obstacle against the downflow and reduces the strength of the horseshoe vortex. For the region below the collar, the strength of the downflow and the strength of the horseshoe vortex are reduced.

**Figure 1:**
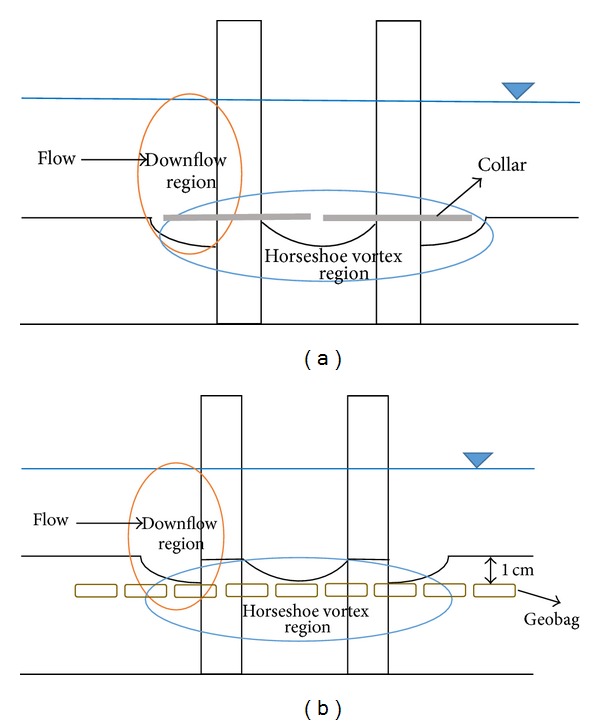
Scour around a pile protected by (a) collar and (b) geobag below sand.

Using a geobag is one of the armoring countermeasures to control the scour around bridge piers. Placing a geobag layer locally around the pier increases the hydraulic resistance against downflow and the horseshoe vortex.  Figure 1 shows the vortex areas around a bridge pier in the presence of a collar and a geobag.

## 3. Experiments and Procedures

### 3.1. Experimental Setup

**Figure 2:**
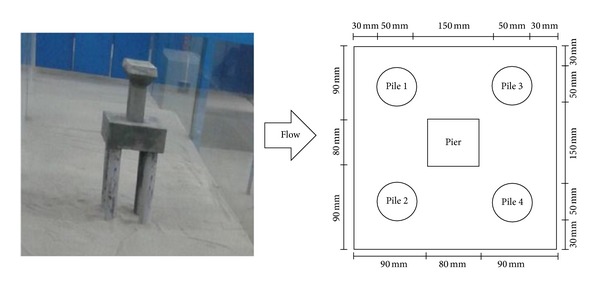
Bridge piers model in laboratory.

The experiments were conducted in a rectangular tilting flume that was 16 m long, 1 m wide, and 1 m deep with a constant longitudinal slope of 0.001. The flume was located in the Hydraulics Laboratory at the University of Malaya. The working section, with a length of 4.4 m, was located at the center of the flume with a control block which was the span that the physical model was located in. The flume was filled with sediment height of 200 mm. Uniform sediment particles with diameters of 0.8 mm were used to fill the flume to depths of 200 mm in the control block and 50 mm outside the control block. An 80 mm wide concrete pier with piles of diameter 50 mm was used as a model of bridge with its substructure. To avoid wall effects on the rate of scour, the maximum diameter of the pier or pile was set to 10% of the width of the flume (2∗5 cm) based on Chiew and Melville [38] recommendations.  Figure 2 shows the geometry of the bridge's substructure.

To obtain the maximum scour depth in clear-water conditions, experiments were performed using uniform sediment (*σ*_*g*_ < 1.5) with flow-intensity values slightly less than the threshold condition of sediment movement (0.9 < *U*/*U*_*c*_ < 1) and *B*/*D* ≥ 10, where *B* is the width of the flume and *D* is the diameter of the pier or pile. These values were chosen so that the side-wall (or blockage) effect attributable to the presence of the pier could be neglected [38]. Noncohesive, uniform sediment with a median particle size of 0.8 mm was used as the bed material, and the geometric standard deviation of the particles, *σ*_*g*_, was equal to 1.29. The critical shear velocity of the bed materials (*U*_c_*) was determined using Shields' diagram, and the critical flow velocity (*U*_*c*_) for sediment entrainment was determined based on the expressions given by Melville and Coleman [3]. The experiments were performed under clear-water conditions at a threshold flow intensity of *U*/*U*_*c*_ = 0.95, where *U* is the average velocity of the approach flow. Therefore, the flow velocity in all experiments always was set to 0.345 m/s. For all of the tests, the relative flow depth used was 35 cm. The water flowing in the flume had to be deep enough to ensure that the depth of the scour hole would not be affected by the flow depth (*y*/*D* > 3.5) [38]. The depth of flow and flow velocity were controlled by the tailgate, which was located at the end of the flume.

**Figure 3:**
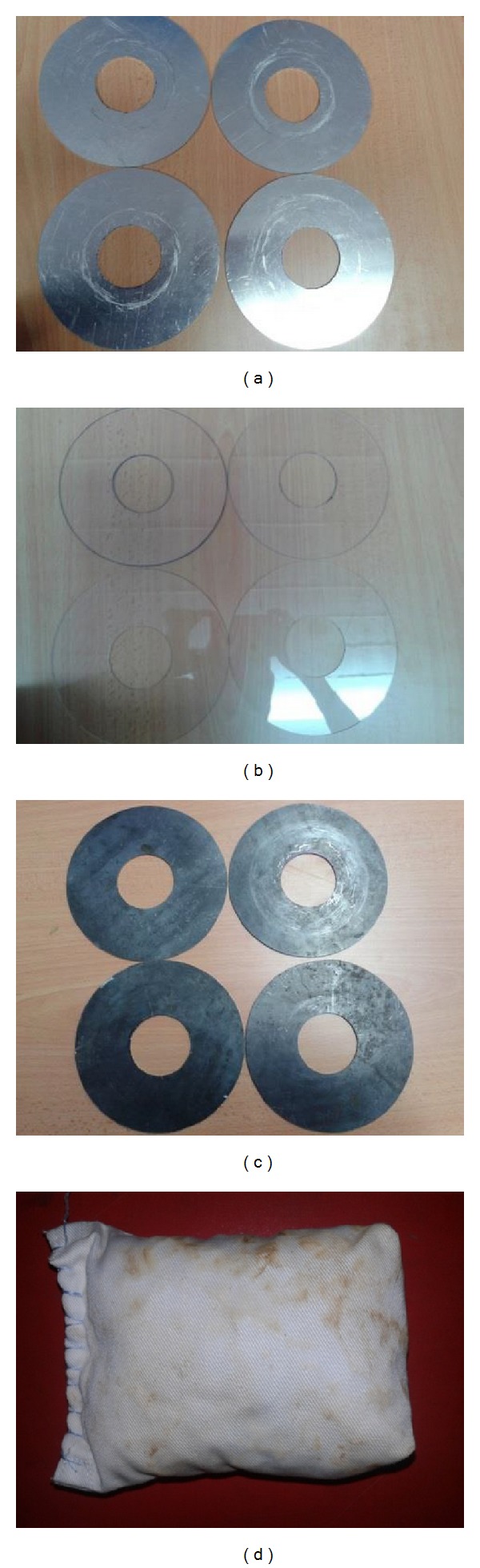
Used countermeasures in laboratory; (a) aluminum collar, (b) Perspex collar, (c) steel collar, and (d) geobag with crushed concrete containing oil palm shell.

The velocity was measured using an electromagnetic current velocity meter and WinLabEm software. A measuring tape was placed at the bridge pile to measure the scour depth in front of the piles. Tests were conducted for seven different installations of the countermeasures as shown in Figure 3.

The same velocity and flow level were used for each test. The development of scouring around the bridge pile was investigated in the first experiment. Then, the three different types of collars were installed for runs two, three, and four, respectively. The width and elevation of the collars were chosen based on previous studies. In the last experiment, the geobag was positioned around the pile 10 mm below the sediment level of the bed. The dimensions of the geobag were calculated based on Pilarczyk [39] equation (1) for scour protection around bridge abutments and piers [27]. From this, the thickness of the geobag, *D*_*B*_, was estimated. The aerial extent should exceed *D*_*B*_. The general form of Pilarczyk's relationship for the thickness of the geobag is presented in (1) [39]:

(1) $$\begin{array}{c} D_{B}=\frac{0.035}{\left(S_{\text{SB}}-1\right)}\frac{\Phi }{\vartheta_{C}}\frac{K_{T}K_{h}}{K_{\text{sl}}}\frac{U^{2}}{2g}, \end{array}$$

where *S*_SB_ is the specific gravity of the geobag, *U* is the depth-average mean velocity, *g* is the acceleration of gravity, Φ is the stability parameter, *ϑ*_*C*_ is the critical value of Shields' parameter for particle (geobag) entrainment, *K*_*T*_ is the turbulence factor, *K*_*h*_ is the depth parameter, and *K*_sl_ is the slope parameter.

The collars were made of steel, aluminum, and Perspex, and they were 2 mm thick; the geobag measured 25 × 105 × 61 mm. Wider collars are more effective, but the construction of collars that are more than three times wider than the diameter of the pile is considered to be impractical. Also, the efficiency of a collar increases at lower elevations since less flow can penetrate below it [20]. The percent efficiency of a countermeasure in terms of scour depth reduction, *r*_ds_, was calculated from

(2) $$\begin{array}{c} r_{\text{ds}}=\frac{d_{\text{so}}-d_{\text{sp}}}{d_{\text{so}}}100\left(\%\right), \end{array}$$

where *d*_so_ and *d*_sp_ are the maximum scour depth in proximity to the unprotected and protected pile, respectively. This was measured at the end of each test [30, 31].  Table 1 describes the experiments that were conducted.

The scour depths at the bridge piles were recorded in three stages. They were recorded 10 times at 1 min intervals. Then, beginning at 10 min, they were recorded 10 times at 10 min intervals. Beginning at 110 min, they were recorded five times at 100 min intervals for every interval of 100 minutes (1 hour and 40 minutes). To this point, the scour depth had been recorded 250 times, and it was recorded one final time after 24 h. The scour depth was measured for every bridge pile.

The criterion for equilibrium scour time in this study was based on recommendation of Melville and Chiew [40] and Sheppard et al. [41], because the scour depth does not change by more than 5% of the diameter of the pier over a period of 24 h [40, 41]. In the initial stage, the tests were run for approximately 48 hours. The observations showed that, in the initial experiment that had duration of 48 h, the scour depth did not change more than 5% of the diameter of the pier over a period of 24 h. Therefore, the duration of all experiments was set to 24 h.

## 4. Results and Discussions

### 4.1. Scour Reduction due to Single Countermeasure

**Figure 4:**
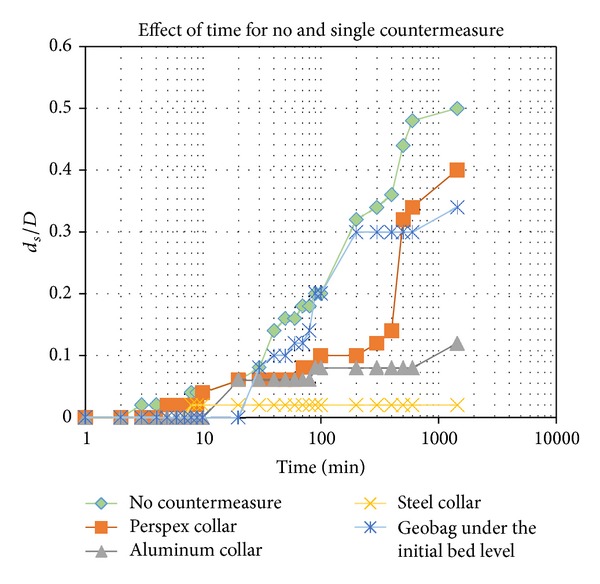
Dimensionless scour depth versus time, for the following scenarios: with countermeasure and without countermeasure (Pile 2).

Figure 4 shows the scour depth for substructure of the bridge's piles versus time for no countermeasure, a Perspex collar, a steel collar, an aluminum collar, and a geobag containing crushed concrete and palm shells. The result of the test of an unprotected pile showed a continuous increase in the scour depth. This was because the unprotected pile had a horseshoe vortex that moved actively. The horseshoe vortex in front of the piles caused a deeper scour depth.

Based on Kumar et al. [23] findings, it is more effective to install a wider collar at a lower elevation, closer to the surface of the riverbed. Consequently, the dimension of the collar was three times the dimension of the piles. Zarrati et al. [42] found that two independent collars placed in line with each other had better efficiency than a continuous collar around both piles. In this experiment, we also used the piles in a line and independent collars. The steel collar countermeasure with a length of 150 mm and thickness of 2 mm provided an acceptable result. This is because the steel collar, which weighed 146 g, was heavier than the other countermeasures, that is, 21 g for Perspex and 49 g for the aluminum collar. The weight of the countermeasure affects the horseshoe vortex so that the vortex cannot move actively, thereby decreasing the depth of the scour. The scour depth also decreased over time when a collar countermeasure was used. Thus, a heavier countermeasure will have a more significant effect on the scour depth.

A geobag filled with crushed concrete is an alternative to the use of a sandbag as a countermeasure to bridge scour. It is made from recycled concrete, which makes it an environmentally friendly approach. Crushed concrete in a geobag is a better solution than using crushed concrete as riprap. This is due to the fact that the dimensions of crushed concrete must be designed with restrictions, while the dimensions of a geobag are designed by an engineer in a more flexible way [28]. A mixture of crushed concrete with oil palm shells has a high density, so the bonding between the material in the oil palm shells and the crushed concrete reduces the scour depth. The horseshoe vortex that occurs above the geobag moved faster than it did when a collar countermeasure was used. The geobag was installed 10 mm below the level of the sediment. Yoon [43] found that *Y*/*D* = 0.2 was an effective ratio for better protection of the bridge's pier, where *Y* is the level of the top surface of gabion below the original level of the bed and *D* is diameter of the pier or pile.

### 4.2. Scour Reduction due to Combined Countermeasure

**Figure 5:**
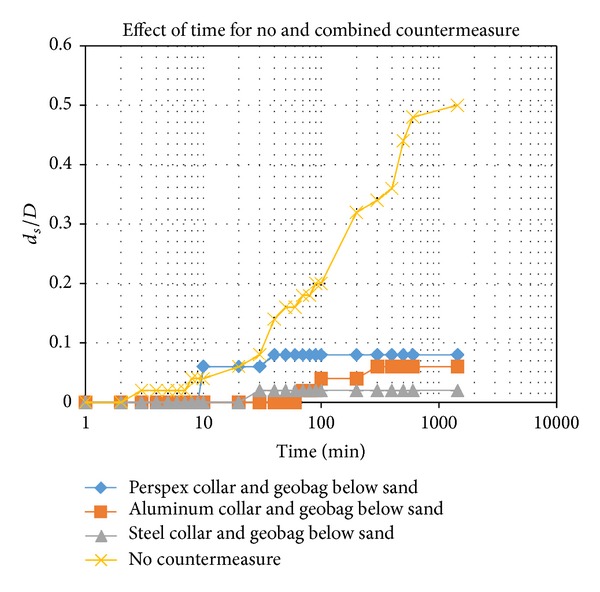
Dimensionless scour depth versus time, for the following scenarios: with combined countermeasure and without countermeasure (Pile 2).

Figure 5 shows the results for no countermeasure and for the combined countermeasures of a Perspex collar and a geobag, an aluminum collar and a geobag, and a steel collar and a geobag below the sediment.

There is a great difference between no countermeasure and the combination of countermeasures surrounding the pile bridge. The most effective result achieved from the combination of countermeasures occurred when a steel collar and a geobag were used. Because steel was heavier than the other collars, it affected the movement of the horseshoe vortex in the front of the pile. Also, the pile is covered with geobag 10 mm below the sediment. This position protected the pile from the scouring effect. The result for all three runs gradually increased, but the increase when there was no countermeasure occurred much more rapidly. The least scour depth was obtained from the combination countermeasure using a Perspex collar and a geobag. This result was better than using only the Perspex collar. As mentioned, the protection provided by the geobag below the sand had an inhibitory effect on scouring. The use of a combination countermeasure gave better result than using a single countermeasure.

The scour reduction achieved for the front and rear piles caused by independent collar and geobag using different types of countermeasures is shown in Table 2. There was a 96% reduction in the scouring of the rear pile when the combination of a steel collar and a geobag was used, indicating that the combination of a steel collar and a geobag was more effective at reducing scour than the other countermeasures. Scouring in the combination of steel collar and geobag occurred at 30 min, whereas it occurred in 8 min for the single steel collar. The better efficiency of the steel collar might be due to a weaker downflow at the upstream face of the rear pile since the steel was heavier than the other materials. The maximum scour depth was observed at the upstream face of the front pile for all tests.

## 5. Conclusions

Collars and geobags are used at bridge piles to reduce local scouring. A collar and a geobag divert the downflow and protect the riverbed from direct impact. In this paper, we reported the results of our study of the application of collars at piles and geobags around the piles. Collars were installed at the sediment bed level with an effective width that was three times the diameter of the pier for all experiments of collar countermeasures and with a geobag located around the piles. Experiments were conducted over a 24 hr period. All tests were conducted at the threshold of motion of the bed material, at which the maximum depth of the scour hole was expected. With collars installed at the streambed level, there was no sign of scouring or the horseshoe vortex at the upstream face of the piers at the beginning of the experiment. In contrast with unprotected piers, in all of the experiments, scouring started from downstream of the piers due to the action of wake vortices. Then the scour holes were extended upstream, and they undermined the collars. Crushed concrete mixed with oil palm shells has greater strength, so the bonding between the material in the oil palm shell and the crushed concrete decreased the voids and thus reduced scouring. The reduction of scour on the rear piles by the steel collar and the combination of the steel collar and a geobag was 96%, indicating that the combination of a steel collar and a geobag was more effective than any of the other countermeasures.

## Figures and Tables

**Table 1:** Summary of experiments.

Run number	Type of countermeasure	Flow depth, *y* (cm)	Flow velocity, *U* (m/s)	Test time, *T* (hr)
1	None	35	0.345	24
2	Perspex collar	35	0.345	24
3	Aluminum collar	35	0.345	24
4	Steel collar	35	0.345	24
5	Geobag	35	0.345	24
6	Perspex collar and geobag under the initial bed level	35	0.345	24
7	Aluminum collar and geobag under the initial bed level	35	0.345	24
8	Steel collar and geobag under the initial bed level	35	0.345	24

**Table 2:** Percentage of reduction of scour depth at the rear pile.

Number	Type of countermeasure	Scour reduction after 24 h, *r*_ds_ (%)
1	None	—
2	Perspex collar	20
3	Aluminum collar	76
4	Steel collar	96
5	Geobag	44
6	Perspex collar and geobag below sediment	86
7	Aluminum collar and geobag below sediment	88
8	Steel collar and geobag below sediment	96

## Notations

*B*: Flume width
*D*: Pile or pier diameter
*y*: Flow depth
*σ*_*g*_: Geometric standard deviation of particles
*U*_*c*_*: Critical shear velocity
*U*_*c*_: Critical flow velocity
*U*: Average approach flow velocity
*U*/*U*_*c*_: Threshold flow intensity
*S*_SB_: Specific gravity of the geobag
*U*: Depth-average mean velocity
*D*_*B*_: Geobag thickness
*g*: Gravity acceleration
Φ: Stability parameter
*ϑ*_*C*_: Critical value of the Shields' parameter for particle (geobag) entrainment
*K*_*T*_: Turbulence factor
*K*_*h*_: Depth parameter
*K*_sl_: Slope parameter
*d*_so_: Maximum scour depth in unprotected conditions
*d*_sp_: Maximum scour depth in protected conditions
*r*_ds_: Scour depth reduction (%)
*T*: Test time
*Y*: Level of the top surface of geobag below the original bed level.
